# Highly sensitive non-enzymatic electrochemical glucose sensor based on dumbbell-shaped double-shelled hollow nanoporous CuO/ZnO microstructures

**DOI:** 10.1038/s41598-020-79460-2

**Published:** 2021-01-11

**Authors:** Zahra Haghparas, Zoheir Kordrostami, Mohsen Sorouri, Maryam Rajabzadeh, Reza Khalifeh

**Affiliations:** 1 Department of Electrical and Electronic Engineering, Shiraz University of Technology, Shiraz, Iran; 2 Department of Chemistry, Shiraz University of Technology, Shiraz, Iran

**Keywords:** Biochemistry, Biotechnology

## Abstract

A high-performance non-enzymatic glucose sensor based on hybrid metal-oxides is proposed. Dumbbell-shaped double-shelled hollow nanoporous CuO/ZnO microstructures (CuO/ZnO-DSDSHNM) were prepared via the hydrothermal method using pluronic F-127 as a surfactant. This structure is studied by various physicochemical characterizations such as scanning electron microscopy, X-ray diffraction spectroscopy, inductively coupled plasma atomic emission spectroscopy, elemental mapping techniques, X-ray photoelectron spectroscopy, and transmission electron microscopy. This unique CuO/ZnO-DSDSHNM provides both a large surface area and an easy penetrable structure facilitating improved electrochemical reactivity toward glucose oxidation. The prepared CuO/ZnO-DSDSHNM was used over the glassy carbon electrode (GCE) as the active material for glucose detection and then coated by Nafion to provide the proposed Nafion/CuO/ZnO-DSDSHNM/GCE. The fabricated glucose sensor exhibits an extremely wide dynamic range from 500 nM to 100 mM, a sensitivity of 1536.80 µA mM^−1^ cm^−2^, a low limit of detection of 357.5 nM, and a short response time of 1.60 s. The proposed sensor also showed long-term stability, good reproducibility, favorable repeatability, excellent selectivity, and satisfactory applicability for glucose detection in human serum samples. The achieved high-performance glucose sensing based on Nafion/CuO/ZnO-DSDSHNM/GCE shows that both the material synthesis and the sensor fabrication methods have been promising and they can be used in future researches.

## Introduction

Electronics and Chemistry researchers have to work jointly to make fast and sensitive electrochemical glucose sensors realizable. This collaboration is vital to detect a silent killer called diabetes which might develop dangerous complications like damages in the kidney, nerves, eyes, and cardiovascular problems^1^. Extremely elevated blood glucose level is found to be one of the leading causes of death in the world. A new study in 2017 estimates that diabetes is the 3rd (not 7th as reported by CDC) leading cause of death^2^. Since most diabetics do not recognize early signs of diabetes and since ignoring it has cruel costs, regular monitoring and sensitive detection of the blood glucose level is vital to diagnose and control this life-threatening illness.

Today, most of the commercially available electrochemical glucose biosensors have been fabricated with glucose oxidase enzyme, which is the most preferred enzyme for blood glucose detection^3,4^. They have some advantages such as sensitive amperometric response, selectivity, and direct point care assays^4,5^. However, researchers are never satisfied with the current technology and thus trying to replace the enzymatic glucose sensors with enzyme-free sensors. The reason is that the enzymatic sensors suffer from several enzyme drawbacks such as enzyme denaturation, low stability, weak enzyme immobilization on the electrode, and complex enzyme purification process^3,5−7^. In fact, the unavoidable drawbacks of enzymes, are important obstacles, that have restricted the progress in enzymatic glucose sensors^8^.

Enzyme-free glucose sensors seem to be promising alternatives that eliminate the problems associated with the enzymes, however, current non-enzymatic glucose sensors still require noticeable improvements in the sensitivity and selectivity to be used for commercial purposes^4^.

Since the electrode surface plays an important role in designing non-enzymatic glucose sensors, synthesizing more sensitive materials with new morphologies that improve all the sensor performance parameters without sacrificing any of them is currently an attractive topic of research and development^9^. Despite the exciting progresses achieved in fabricating micro and nano structures to date, it is still desirable yet challenging to synthesis high-quality complex multi-shelled hollow spheres including more than one metal oxides^10−13^.

Qi et al. have reviewed the properties and applications of multi-shelled hollow micro/nano structures which shows that the additional shells can improve the performance because they provide additional sites for interactions^14^. The micro/nano hollow structures provide advantages such as large contact area between electrode and electrolyte, high permeability, more active sites, low density, and shorter path of electron transfer which are very interesting for sensor applications^9,15−17^. Hollow structures prevent the agglomeration of nanoparticles owing to their interior voids and this helps in better accommodation of volume changes in successive electrochemical measurements^9,17−19^.

The outstanding catalytic activity of transition metal oxides such as ZnO and CuO in alkaline solutions toward glucose detection have been reported numerously in previous researches^20−23^. Unlike expensive noble metals these transition metal oxides are cost-effective, widely used for sensor fabrication, and less affected by interfering species^8,9,24^. ZnO and CuO are inexpensive semiconductors, non-toxic and both have excellent electrochemical and catalytic properties. It is possible to synthesize different morphologies of ZnO and CuO by using simple methods and low temperatures. By tuning the process parameters, the morphologies of these metal oxides can be controlled. These parameters include the carbon template source, the concentration of metal salts, and the time of the hydrothermal process.

Because of the variety of morphologies and high surface area to volume ratio of ZnO and CuO nanostructures, they are known as promising materials and a universal choice of researchers for building high-performance glucose sensors^6^.

Also, ZnO nanostructures with favorable characteristics such as large surface area, high crystallinity, and good electrical properties can be modified by other nanomaterials to provide enhanced sensing properties.

On the other hand, the CuO nanostructures have unique advantages such as the high surface area, low density, presence of active sites even in the interior space, and favorable permeation^25^. ZnO is a low-cost, broad bandgap energy (3.37 eV), high-mobility, non-toxic mineral compound considered as an n-type semiconductor. CuO is a p-type semiconductor with a narrow bandgap (1.2–1.9 eV) that can enhance the electrochemical properties in combination with ZnO^26,27^. Because of their excellent electrochemical behavior, most of the works have used one of the CuO or ZnO nanostructures individually to develop their biosensors. More recently, hybrid nanostructured materials based on ZnO have been proposed^6,22,27−31^. They exhibit larger surface-to-volume ratios which in turn activates the electrocatalytic properties of the materials further. Besides, because of the high electron mobilities in ZnO based hybrid materials, the transfer of the electrons to the supporting electrodes becomes easier and faster^6^.

Some researchers have studied the hybrid and hollow structures toward glucose detection by designing new metal oxides morphologies^9^. Recently, non-enzymatic glucose sensors have been fabricated using Cu/Cu_2_O/CuO^32^, Cu_2_O^33^, core-sheath nanocarbon^20^, double-shelled CuCo_2_O_4_^34^, CuO/Cu_2_O composite^35^, Cu_x_S^36^, NiS^37^, NiO^38,39^, CO_3_O_4_^40^ hollow spheres, hollow CuO/Pani^41^, carbon nanofibers^42^, cubic CuO_x_/NiO_y_ nanocomposite^9^, cubic NiO porous architecture17, and NiCo_2_O_4_ Nanocages^43^. Ahmad et al. directly grew ZnO nanorods (NRs) on the FTO electrode surface (ZnO NRs/FTO) using a low-temperature hydrothermal method and functionalized them with CuO (CuO-ZnO NRs/FTO electrode) to enhance the electrochemical activity for glucose detection. The electrochemical sensing of CuO-ZnO NRs/FTO electrode exhibited a high sensitivity of 2961.7 μA.mM^−1^.cm^−2^, linear range up to 8.45 mM, and a low limit of detection (LOD) of 0.40 μM^6^. Zhou et al. obtained a three-dimensional (3D) porous ZnO–CuO hierarchical nanocomposites (HNCs) through one-step electrospinning together with ordinary hot press and calcination process. The optimum ZnO–CuO HNCs electrode presented a high sensitivity of 3066.4 µA mM^−1^ cm^−2^, a linear range up to 1.6 mM, and a practical LOD of 0.21 mM^28^. Ridhuan et al. fabricated ZnO nanorods on a seeded substrate which were prepared by the sol–gel method, wherein a seed layer was coated onto ITO substrates. The Nafion/GOx/ZnO NRs/ITO electrode which was used as a modified electrode for glucose detection displayed a linear response to glucose ranging from 0.05 to 1 mM, with a sensitivity of 48.75 μA/mM^5^.

In this paper, a hybrid material has been achieved via the hydrothermal method based on the combination of two transition metal oxides (CuO/ZnO) in a form of dumbbell-shaped double-shelled hollow nanoporous microstructures and also used as a non-enzymatic high sensitivity glucose biosensor. Our sensor benefits from enhanced sensing characteristics due to the appropriate combination of two kinds of semiconductor oxides. The excellent electrochemical performance of CuO/ZnO-DSDSHNM as a non-enzymatic glucose sensor was verified when outstanding sensor parameters were obtained. As another step in the enrichment of our scientific report, we also fabricated pure ZnO and CuO hollow spheres separately and showed that the sensing ability of the ZnO-CuO hybrid material is much higher than them.

## Results and discussion

### Structural and morphological properties

**Figure 1:**
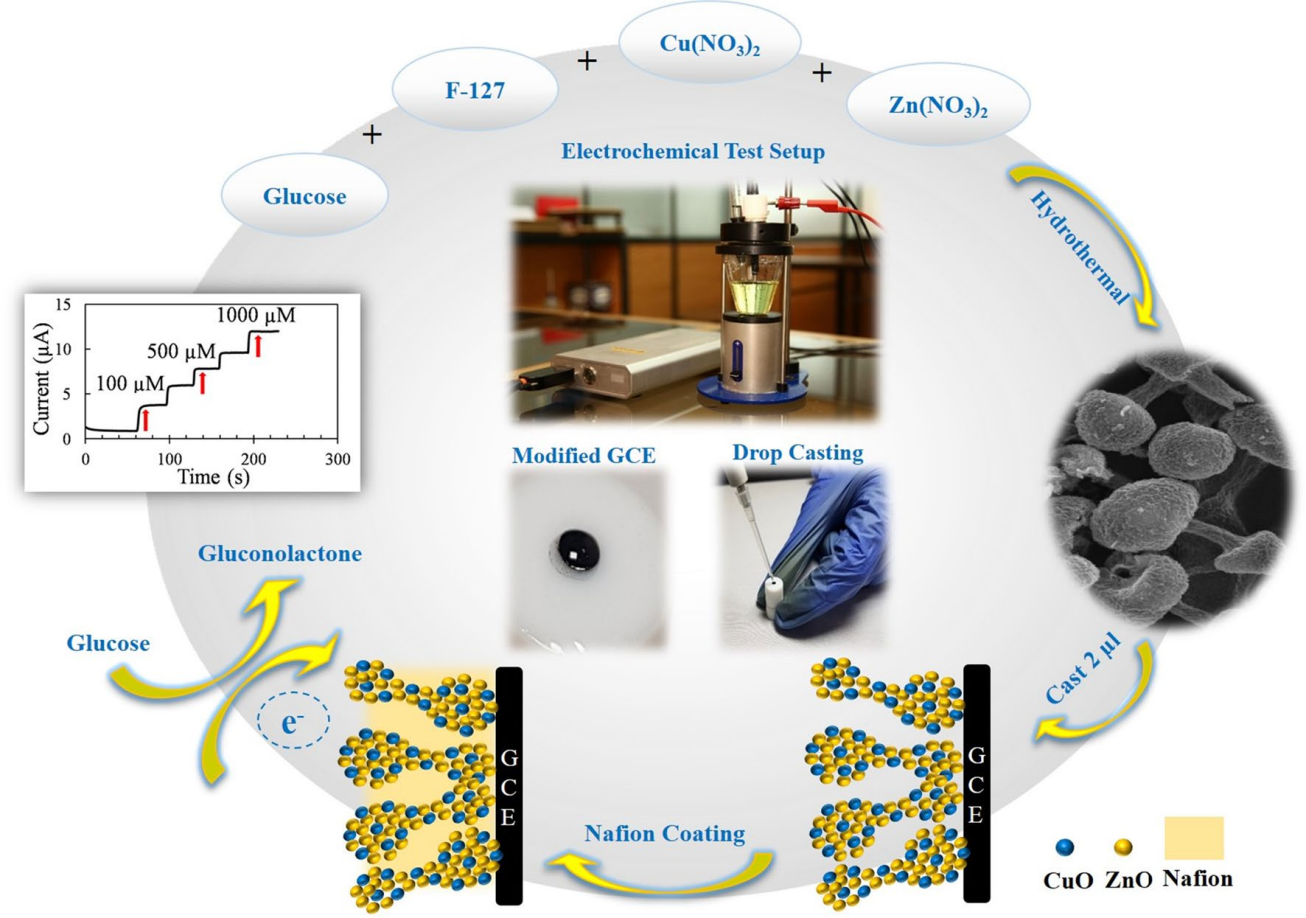
Schematic illustration of the construction and application of CuO/ZnO-DSDSHNM non-enzymatic biosensor for the determination of glucose in human blood samples.

**Figure 2:**
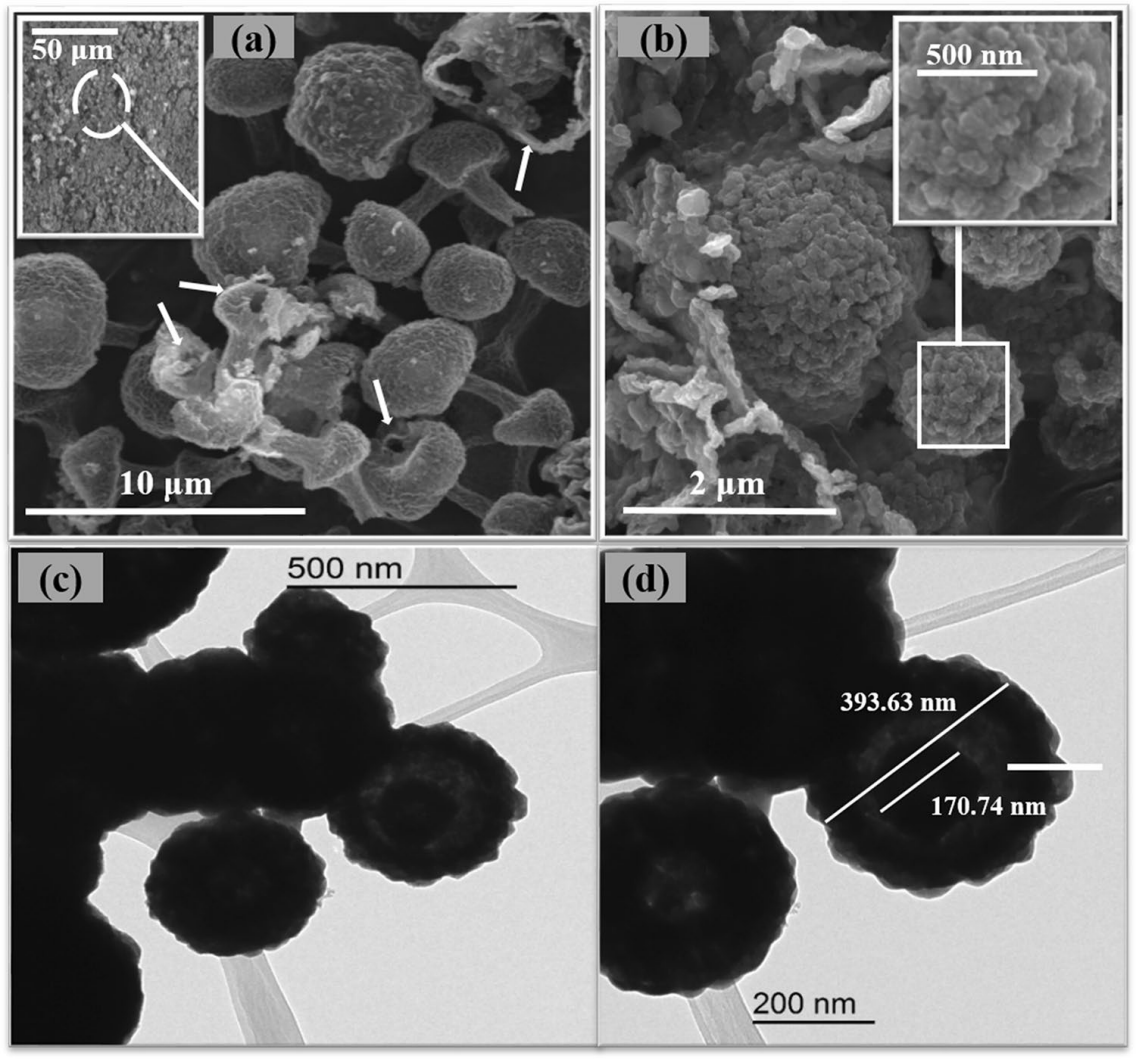
(**a**) And (**b**) typical SEM micrographs of CuO/ZnO-DSDSHNM, (**C**), and (**d**) TEM images of CuO/ZnO-DSDSHNM.

**Figure 3:**
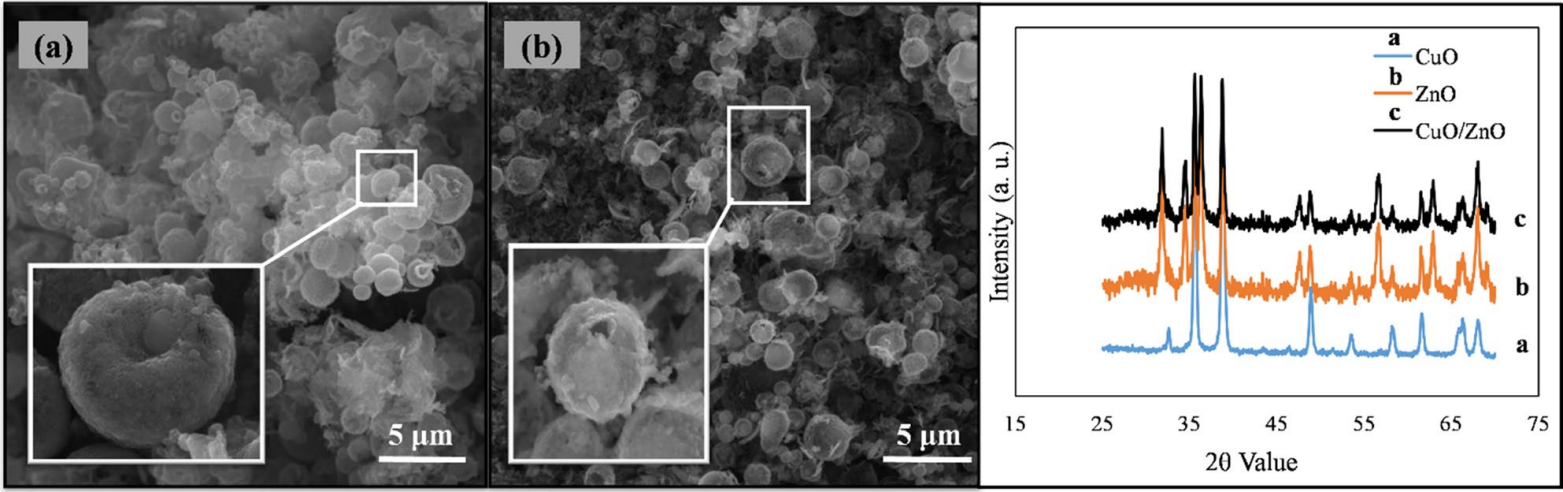
SEM micrographs of (**a**) CuO-MS and (**b**) ZnO-MS, (**c**) XRD patterns of as-synthesized CuO/ZnO-DSDSHNM.

The proposed non-enzymatic glucose sensor was designed based on Nafion/CuO/ZnO-DSDSHNM/GCE. As brought into the methods section, the unique structure of CuO/ZnO-DSDSHNM was achieved via the hydrothermal method. The CuO/ZnO-DSDSHNM has been used as the sensing material which has been deposited on a glassy carbon electrode (GCE) by using a casting procedure. The schematic representation of the sensor construction steps is summarized in Fig. 1. The SEM of as-synthesized CuO/ZnO-DSDSHNM is shown in Fig. 2a,b. As the main morphology of the material particles, the dumbbell shape structures are clearly observed. In many cases, the size of the heads at each side of the dumbbell is not equal (asymmetrical dumbbell). The hollow nature of the synthesized structure is signified at the inset in Fig. 2a. The hollow nature of these particles could be directly related to the presence of carbon microspheres as a hard template. During the hydrothermal process, the carbon microspheres (Formed in situ from glucose precursor) served as the template for the fabrication of the final hollow multi-shelled nanostructure. The removal of the template during the calcination step under the air atmosphere is responsible for the formation of a hollow multi-shelled nanostructure by the strategy of “hard template synthesis”. Using the oxygen-containing air as the calcination atmosphere causes the conversion of the template to CO and facilitates the removal of the carbon template. According to the SEM micrograph at the inset of Fig. 2b, the nanostructured details of each particle could be observed. In addition, the integration of nanoparticles to form the final micron-sized particles has rendered the nanoporous nature of the synthesized particles which is also clear in the SEM micrograph. The formation of the nanoporous surfaces could be attributed to the presence of pluronic F-127 as a surfactant in the synthesis procedure. The holes over some particles are remarked by arrows in Fig. 2a which implies the hollow nature of these particles. Further observation utilizing transmission electron microscopy (TEM) confirmed the results obtained from the analysis of the SEM images. The TEM images indicate the double-shelled structures of CuO/ZnO. As can be seen in Fig. 2c,d, the dumbbell-shaped CuO/ZnO structures are composed of two layers. The inner shell diameter is 170.74 nm and the outer shelled diameter is 393.63 nm (Fig. 2d). Figure 3a,b represent the SEM images of separate CuO and ZnO microspheres (CuO-MS and ZnO-MS), respectively. According to the SEM micrograph of the CuO microstructures, the formation of bean-like particles with an average diameter of 1.50 µm from small nanoparticulate blocks could be observed (Fig. 3a)^44^. In the case of ZnO microstructures (Fig. 3b) it has been shown that they have an average size of 1.50 µm. Also, the nanoparticulate, as well as the hollow nature of the synthesized structures, has been observed. The X-ray diffraction spectroscopy (XRD) of CuO-MS, ZnO-MS, and CuO/ZnO-DSDSHNM are represented in Fig. 3C. The XRD pattern of CuO corresponds to the JCPDS # 00–045-0937. The diffraction peaks at 2θ values of 32.60, 35.60, 38.80, 48.92, 53.68, 58.40, 61.68, 66.48, 68.20, 72.72 and 75.28 could be indexed as (− 110), (002), (111), (− 202), (020), (202), (− 113), (− 311), (− 220), (311) and (− 222) planes, respectively^44^. According to diffractogram b, the ZnO XRD planes at 2θ values of 31.80, 34.40, 36.25, 47.50, 56.60, 62.95, 66.50 and 68.05 could be indexed as (100), (002), (101), (102), (110), (103), (200) and (201) planes in correspondence with JCPDS # 00–036-1451. Finally, the XRD pattern of CuO/ZnO-DSDSHNM was illustrated as diffractogram c. It is also obvious that the characteristic peaks of both CuO and ZnO at 2θ values of 34.40, 47.50, 62.95, 66.50 and 68.05 are remarkable in the diffractogram of CuO/ZnO-DSDSHNM. According to obtained XRD peaks, the phase purity of CuO and ZnO are conserved and the corresponding peaks are obvious.

**Figure 4:**
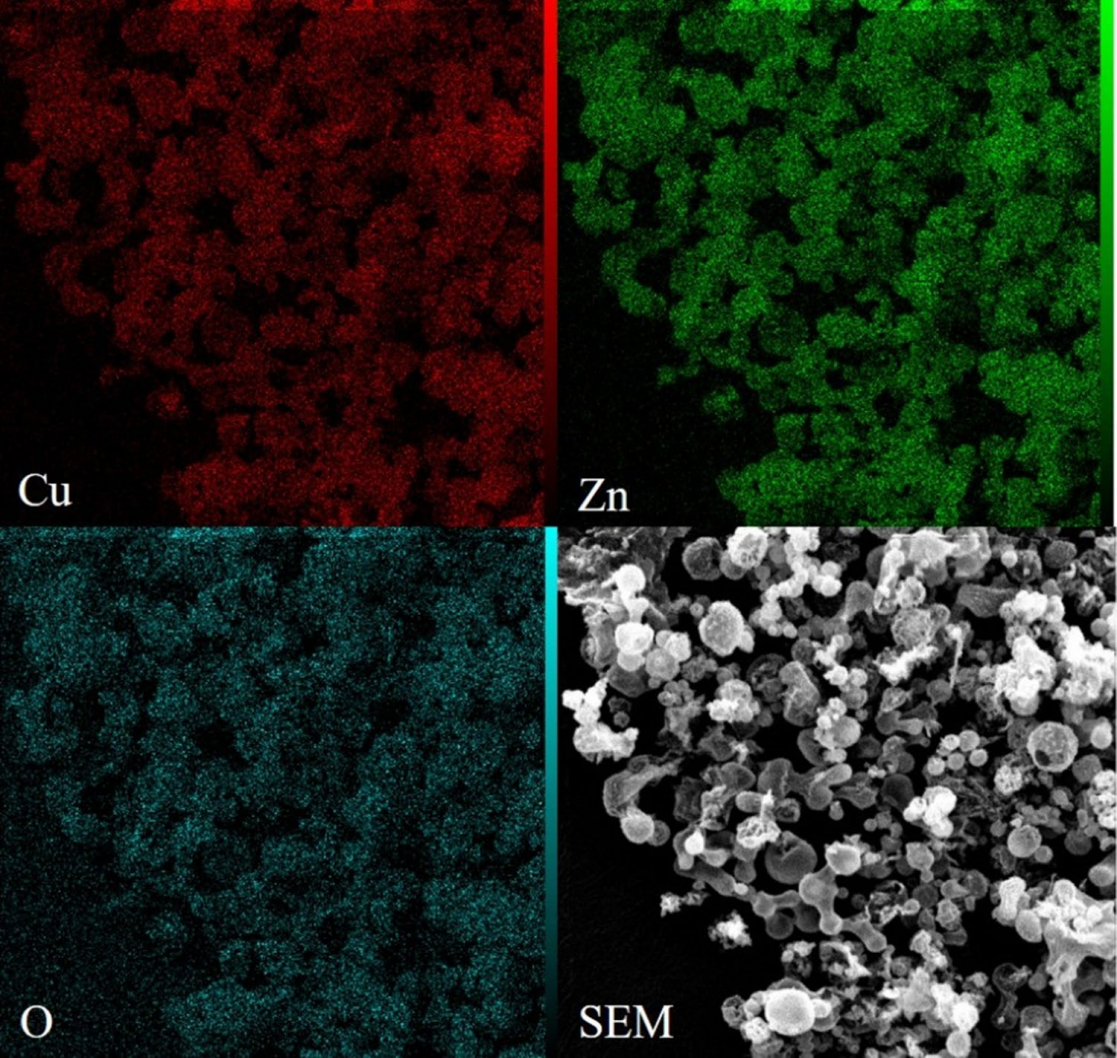
The elemental mapping technique of CuO/ZnO-DSDSHNM.

The inductively coupled plasma atomic emission spectroscopy (ICP-AES) showed that the atomic ratio of Cu over Zn is 1.4 which shows a very close distribution. In addition, the elemental mapping of the CuO/ZnO-DSDSHNM is depicted in Fig. 4. The uniform distribution of Cu (red), Zn (green), and O (blue) over the entire sample could be deduced. It can be seen that the ICP results are in almost good agreement with the elemental mapping results. Conclusively, the homogeneous structure of particles as a composite of CuO and ZnO materials has been verified.

**Figure 5:**
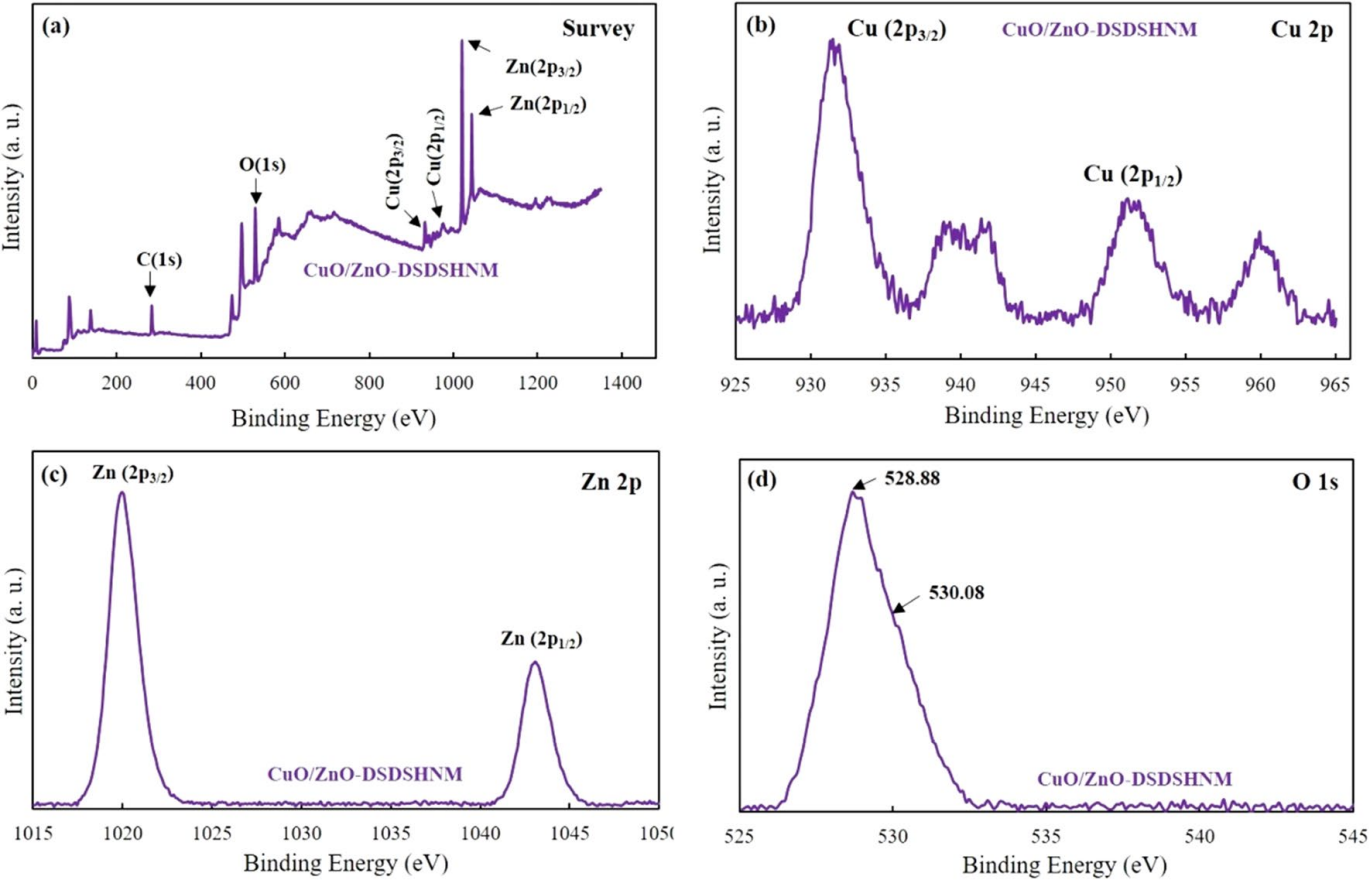
XPS spectra of CuO/ZnO-DSDSHNM showing full scan survey (**a**) and corresponding peaks in the high-resolution spectra for Cu 2p (**b**), Zn 2p (**c**), and O 1 s (**d**).

X-ray photoelectron spectroscopy (XPS) of the samples was studied to further investigate the chemical state of the composing elements and also the surface composition of the CuO/ZnO-DSDSHNM samples. The XPS results have been shown in Fig. 5. The survey spectrum illustrates that Cu, Zn, O, and C are present in the samples which verifies the successful synthesis of CuO/ZnO-DSDSHNM (Fig. 5a). There are two dominant peaks in the spectrum of Cu at 931.68 eV (Cu 2p_3/2_) and 951.58 eV (Cu 2p_1/2_). These peaks can respectively be fitted to Cu^2+^ (930.98 and 950.98 eV) and Cu^+^ (932.18 and 953.38 eV). The presence of the component CuO has been validated by the existence of two satellite peaks at ∼940 and ∼960 eV that correspond to the Cu^2+^ oxidation state (Fig. 5b)^4,45−47^. As can be seen in Fig. 5c, high-resolution Zn 2p spectra of CuO/ZnO-DSDSHNM showed two symmetric peaks. The presence of the Zn in the matrix (in + 2 oxidation state) can be concluded from the peaks at ∼1020.08 eV (Zn 2p_3/2_) and ∼1043.28 eV (Zn 2p_1/2_)^48^. Since the O 1 s peak is asymmetric (Fig. 5d), it can be concluded that the surface is comprised of two components of the oxygen. One of the components located at ∼528.88 eV is attributed to the O^2−^ ions bonded with Zn^2+^ or Cu^+^/Cu^2+^ ions, whereas the second one which is located at ∼530.08 eV belongs to the O^2−^ ions in oxygen-deficient regions. The obtained XPS results verify that the oxygen vacancy defects are present on the surface of the synthesized samples^49−51^.

### Electrochemical properties

**Figure 6:**
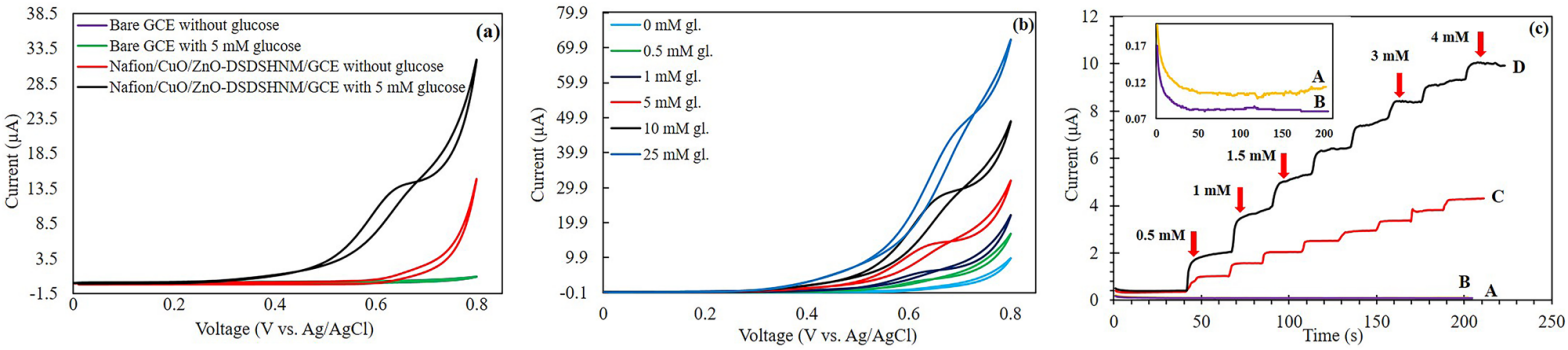
(**a**) The CV responses of the bare and the modified GCE with CuO/ZnO-DSDSHNM at the scan rate of 50 mVs^−1^ in the absence and presence of 5 mM glucose. (**b**)The CVs of Nafion/CuO/ZnO-DSDSHNM/GCE at the scan rate of 50 mVfs^−1^ in 0.5 M NaOH solution at different glucose concentrations. (**c**) i–t curves of (**A**) bare GCE, (**B**) Nafion/ZnO-MS/GCE, (**C**) Nafion/CuO-MS/GCE, and (**D**) Nafion/CuO/ZnO-DSDSHNM/GCE by successive addition of 0.50 mM of glucose to the final concentration of 4 mM. The inset plot of (**c**) is the magnified view of the (**A**) bare GCE and (**B**) Nafion/ZnO-MS/GCE by successive addition glucose in the range of 0.50–4 mM.

The GCE has been modified with the synthesized hollow structures and the Nafion/CuO/ZnO-DSDSHNM/GCE was used as a modified working electrode for measurements. The electrochemical CV tests for different working electrodes were performed in 0.50 M NaOH solution at the scan rate of 50 mVs^−1^ within the potential range of 0 to + 0.80 V. The CVs shown in Fig. 6a illustrate the responses of the bare and modified GCE in the absence and presence of 5 mM glucose. The CVs shown in Fig. 6a illustrates the responses of the bare and modified GCE in the absence and presence of 5 mM glucose. As shown in Fig. 6a, in the absence of glucose, no response can be observed for bare GCE and Nafion/CuO/ZnO-DSDSHNM/GCE. However, when glucose is added, a distinguishable response was revealed, which demonstrated the reasonable performance of CuO/ZnO-DSDSHNM toward glucose detection. The CVs shown in Fig. 6a illustrates the responses of the bare and modified GCE in the absence and presence of 5 mM glucose. As shown in Fig. 6a, in the absence of glucose, no response can be observed for bare GCE and Nafion/CuO/ZnO-DSDSHNM/GCE. However, when glucose is added, a distinguishable response was revealed, which demonstrated the reasonable performance of CuO/ZnO-DSDSHNM toward glucose detection. The CV responses for the CuO/ZnO-DSDSHNM modified GCE to different glucose concentrations have been presented in Fig. 6b. As can be seen, by increasing the glucose concentration from 0 to 25 mM the current has also been increased. As Fig. 6b demonstrates, there are remarkable differences between the CVs which shows that the fabricated sensor is very sensitive to the changes in the glucose concentration and the sensor is reacting very sensitively to the increase in the glucose concentration. In order to examine the behavior of the sensor quantitatively, we performed chronoamperometric tests to determine the calibration curve and other performance parameters. The results of the amperometry tests are shown in Fig. 6c. The electrochemical properties of ZnO-MS, CuO-MS, CuO/ZnO-DSDSHNM, and bare GCE have been studied in Fig. 6c and provided trustworthy chemical information. The amperometry tests were performed in the potential of + 0.60 V by the successive addition of glucose from 0.50 to 4 mM. As can be seen in the inset plot of Fig. 6c, bare GCE and ZnO-MS have no electrochemical activity for glucose detection. CuO-MS modified GCE has a weaker electrochemical activity and a lower sensitivity versus glucose concentration^44^ in comparison to the CuO/ZnO-DSDSHNM. Comparing the responses of CuO and ZnO with our proposed hybrid material CuO/ZnO in Fig. 6c reveals that the CuO response is much larger than ZnO. Although ZnO has no significant response to the glucose in the non-enzymatic detection process, however, it has a strong contribution to the enhancement of electrochemical properties of the sensor when it combines with CuO and the proposed hybrid material (CuO/ZnO-DSDSHNM) is formed^6,28^. The integration of metal oxide semiconductors of p-type and n-type enhances the concentration of charge carriers due to better charge separation. In addition, due to the heterogeneous function, an increase in the stability and catalytic activity is achievable^26,27^. As a result of Fig. 6c, the integration of these two semiconductors provides a more favorable electrochemical activity and outstanding responses in comparison with the Nafion/CuO-MS/GCE electrode.

The mechanism of the nonenzymatic electrochemical oxidation of glucose over CuO/ZnO-DSDSHNM electrode could be explained by the semiconductive nature of CuO/ZnO^52^. Herein, despite several types of research that have introduced Cu(3 +) as the active intermediate for the oxidation of glucose^53^, the formation of holes over the semi-conductive surface of CuO/ZnO is considered responsible.

According to Fig. 6, the voltammetry peak related to the conversion of Cu(2 +) to Cu(3 +) was not observable at CuO/ZnO-DSDSHNM in alkaline electrolyte. Instead, also in the absence of glucose, a plateau around 0.5–0.7 V vs. Ag/AgCl was observable. The capacitive nature of this plateau has been approved previously^52^ which could be attributed to the adsorption of hydroxyl ions in the electrolyte to the surface of CuO/ZnO electrode as a p-type semiconductor. The sharp increase of the current after this plateau also could be attributed to the oxidation of these adsorbed hydroxyl species to O_2_ in the high anodic potentials. Following the presence of glucose in the electrolyte, it seems that this electrochemical O_2_ evolution is amplified and the enhancement of plateau is remarkable. Although the well-shaped peak of glucose oxidation over CuO/ZnO-DSDSHNM was not apparent, but the gradual increase of plateau current by successive addition of glucose concentration was encountered (Fig. 6b). Herein, for better distinguishing of the response to various concentrations of glucose, the chronoamperometric technique was chosen instead of cyclic voltammetry. In addition to the above-mentioned electrochemical evidences, the presence of O vacancies over the electrode surface was previously approved by using XPS analysis (Fig. 5).

Due to the application of the unique semiconductive hybrid nanostructures of CuO/ZnO in the construction of CuO/ZnO-DSDSHNM electrode, the adsorption of OH^−^ species from alkaline electrolyte occurred remarkably. In the absence of glucose, the further oxidation of these adsorbed species caused the sharp current increase at the extreme of the anodic sweep. However, in the presence of glucose, some interactions of the analyte with the electrode surface was expectable^52^. According to interactions of glucose with the electrode surface, the amplification of the oxidation process of OH^−^ species occurred and in turn, the current has been increased. The increase of current was linearly related to the glucose concentration and this has been successfully used for sensing application.

According to Fig. 6c, the ZnO-MS modified electrode has no response to the glucose in the non-enzymatic detection process which has no specific property of redox reaction like CuO, however, the role of ZnO in the proposed sensor is to accelerate the electron transfer, which demonstrates the high performance of CuO/ZnO-DSDSHNM as evidenced in Fig. 6c.

**Figure 7:**
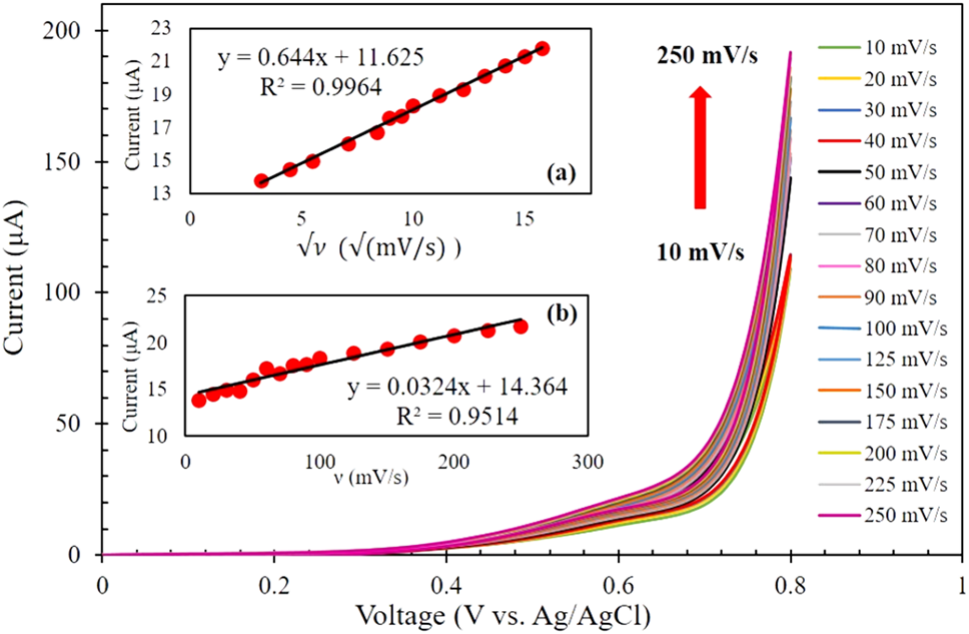
CV responses of CuO/ZnO-DSDSHNM in 0.5 M NaOH at various scan rates ranging from 10 to 250 mVs^−1^ (10, 20, 30, 40, 50, 60, 70, 80, 90, 100, 125, 150, 175, 200, 225 and 250 mVs^−1^) in 10 mM glucose. Inset: corresponding two plots of anodic peaks versus the (**a**) square root of scan rate and (**b**) scan rate.

To survey the kinetics of glucose oxidation on the surface of the Nafion/CuO/ZnO-DSDSHNM/GCE, the effect of the scan rates on the CV currents has been studied for 10 mM of glucose in 0.50 M NaOH solution. Figure 7 shows the CV responses of glucose oxidation at Nafion/CuO/ZnO-DSDSHNM/GCE at different potential scan rates ranging from 10 to 250 mVs^−1^. As illustrated at the inset (a) of Fig. 7, the peak currents exhibited a linear relation versus the square root of the scan rate, demonstrating that the oxidation of glucose at the modified electrode is under the diffusion-controlled behavior. In contrast, as depicted in the inset (b) of Fig. 7, the plot of peak currents versus the scan rate is not favorably linear which indicates the absence of adsorptive behavior^30,31,44^.

### Sensor optimization

The working electrode surface should be modified with CuO/ZnO-DSDSHNM. To enhance the performance of the sensor, it is necessary to cast an optimum amount of CuO/ZnO-DSDSHNM on the GCE surface. Optimizing this parameter will increase the accuracy and sensitivity of the sensor. In order to determine the best concentration of the CuO/ZnO-DSDSHNM, the CV tests were performed at different concentrations of the sample. According to figure S1, the optimum value of this parameter has been selected 5 mg/ml. After that, a coating of Nafion is casted on the surface of CuO/ZnO-DSDSHNM. This coating prevents the falling of nanoparticles from the GCE surface and prevents interfering species from penetrating. It also increases the reproducibility and repeatability of the sensor. Therefore, as another step for optimization, it is necessary to optimize the concentration of the Nafion. Different concentrations of Nafion were prepared and CV tests were carried out in the presence of 5 mM glucose. Figure S2 shows the results of the different Nafion concentrations and accordingly, it is obvious that the optimum concentration is 0.50 wt%. Because the GCE and the CuO/ZnO-DSDSHNM will be oxidized in the absence of the analyte in NaOH solution, several consecutive cycles of the CV measurements have been performed. These cycles were repeated in the absence of glucose until they are overlapped. This way we are sure that by adding glucose, the response of the electrode is only due to the glucose oxidation. The measurements showed that after 10 cycles the proposed sensor behaves in exactly the same way as the last cycle. Thus, to stabilize the results, 10 cycles of CVs must be run before the test starts. After the electrode activation, the best voltage range for improved sensing abilities should be selected. This is one of the most important parameters that is very effective in the performance of the sensor. Based on figure S3, the best results for modified GCE were observed in the range of 0–0.80 V. Optimizing the NaOH concentration results in the maximum production of OH^−^, in which case the oxidation of glucose will be as efficient as possible. According to figure S4, the linear increasing trend of current versus glucose concentration was observed in 0.50 M NaOH solution which also resulted in a higher current. As another design parameter, the best voltage of the glucose oxidation for the amperometry test has to be determined which enables the reliable performance of the sensor. In order to do that, the current–time (i-t) curves of Nafion/CuO/ZnO-DSDSHNM/GCE with the addition of 5 mM glucose in 0.5 M NaOH solution were acquired at different oxidation voltages (Figure S5). According to figure S5, the highest amount of glucose oxidation occurs at a voltage of 0.60 V.

### Non-enzymatic glucose sensing based on CuO/ZnO-DSDSHNM

**Figure 8:**
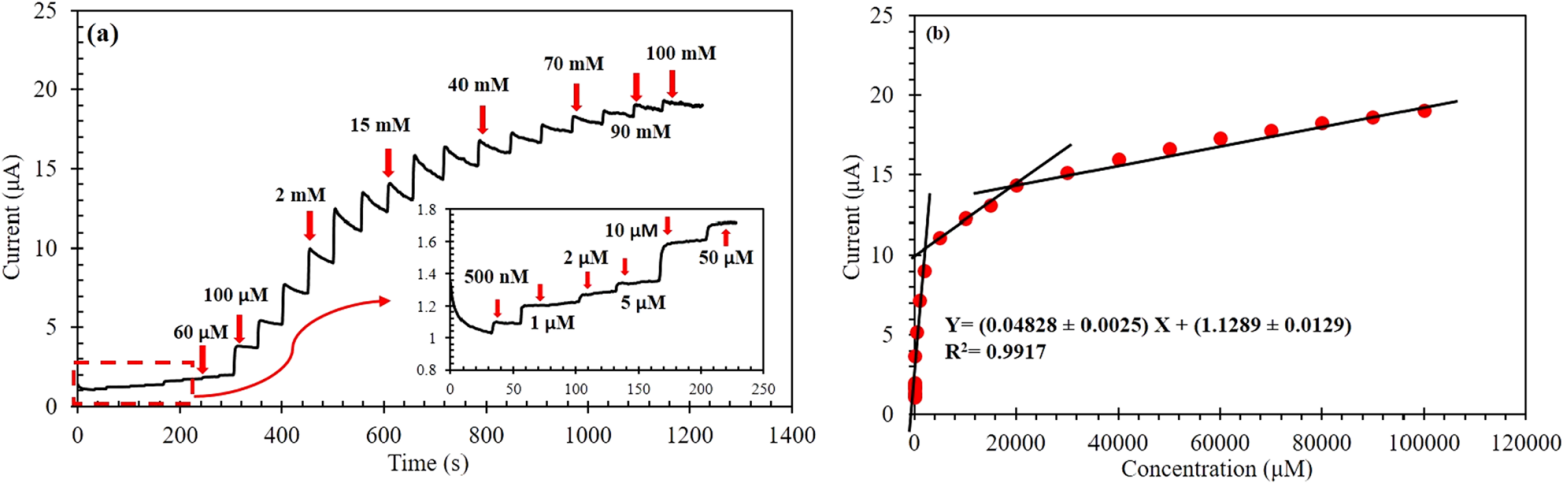
(**a**) Amperometric response of Nafion/CuO/ZnO-DSDSHNM/GCE at 0.60 V in 0.5 M NaOH solution by successive addition of glucose concentration from 500 nM to 100 mM. Inset: the magnified view of the low concentration range of glucose (500 nM–50 μM). (**b**) Calibration plot of the sensor responses versus the glucose concentrations.

For further highlighting and confirming the superior electro-catalytic activity of CuO/ZnO-DSDSHNM, amperometry test was carried out. Figure 8a demonstrates the typical i-t curve of CuO/ZnO-DSDSHNM with successive addition of different glucose concentrations at an optimum applied potential of + 0.60 V under the stirring condition in 0.5 M NaOH solution. By successive additions of various glucose concentrations from 500 nM to 100 mM the consecutive increase in current has been observed. In addition, the inset of Fig. 8a illustrates the magnified view of the low concentration ranges of glucose. Obviously, the first current steps appeared for 500 nM glucose, suggesting the high detection sensitivity of the sensor. The corresponding calibration curve can be described by a power plot (Figure S6) which could be broken into three linear ranges with the following regression equation for the first linear range: I(μA) = (0.04828 ± 0.0025) C(μM) + (1.1289 ± 0.0129) (R^2^ = 0.9917) for 0.5 µM–2 mM glucose (Fig. 8b). The sensitivity can be calculated as the ratio of the slope to the electrode area. The novel electrochemical glucose biosensor was designed, constructed, and optimized so that it has a wide dynamic range from 500 nM to 100 mM which included three linear ranges 500 nM–2 mM, 2 mM–20 mM, and 20 mM–100 mM, and an outstanding sensitivity of 1536.80 ± 79.60 µA mM^−1^ cm^−2^. Upon the addition of 500 nM of glucose, the Nafion/CuO/ZnO-DSDSHNM/GCE reached a steady-state with a very sharp response. It raised to 90% of the response within 1.60 s which is highly favorable comparing with similar sensors which are brought in Table 1. Also, the limit of detection is calculated as 357.5 nM which shows that the sensor has provided a very low detection limit. This indicates that the proposed sensor has a high capability for fast electrocatalytic detection of glucose.

**Table 1:** Performance comparison of the proposed sensor to the previous works.

Electrode	Voltage (V)	Sensitivity (μA.mM^−1^.cm^−2^)	Detection limit (μM)	Dynamic range (mM)	Response time (s)	References
Nafion/CuO/ZnO-DSDSHNM/GCE	0.60	1536.80	0.357	0.0005–100	1.60	This work
Nafion/GOx/ZnO/ITO	− 0.70	48.75	60	0.05–1.00	10	^5^
		3.87		1.00–20.00		
CuO-ZnO NRs/FTO	0.62	2961.70	0.40	0.001–8.45	< 2	^6^
ZnO-CuO HNCs	0.70	3066.4	0.21	0.00047–1.6	5.5	^28^
CuO Nanospheres	0.60	404.53	1	Up to 2.55	–	^54^
CuO/graphene	− 0.05	37.63	5	5–14	1	^4^
GCE/ZnO@NDCS/GOx	0.57	231.7	6.3	0.2–12	3	^30^

The analytical performance of the as-prepared CuO/ZnO-DSDSHNM sensing system for glucose detection is superior to or comparable with many previously enzymatic and non-enzymatic glucose sensor^4,30,31,54^ (Table 1).

### Reproducibility, repeatability, selectivity, and stability tests

**Figure 9:**
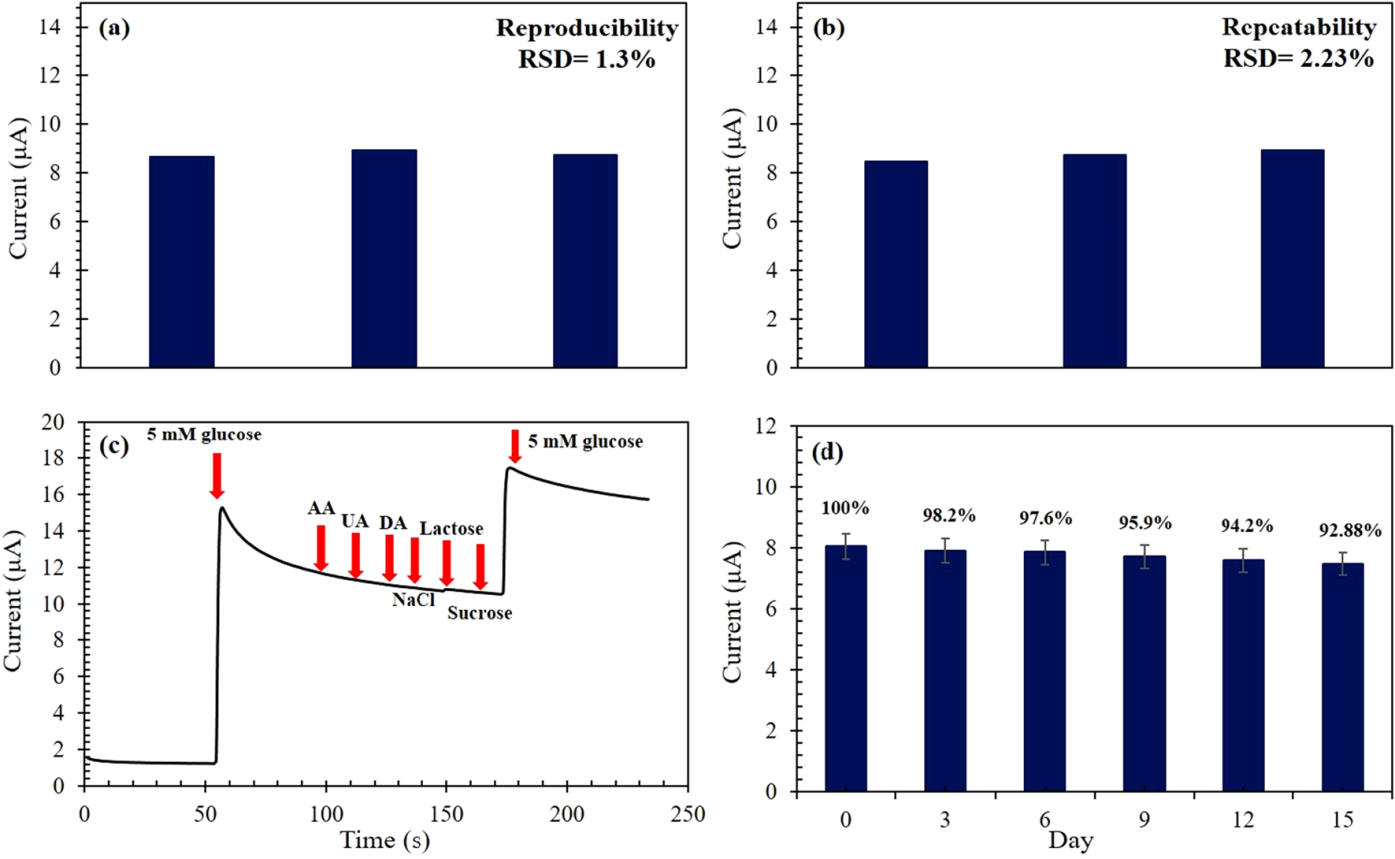
(**a**) Reproducibility, (**b**) repeatability, (**c**) selectivity, and (**d**) stability of Nafion/CuO/ZnO-DSDSHNM/GCE response for 5 mM of glucose.

Reproducibility, repeatability, selectivity, and stability are critical parameters for the investigation of the performance of sensing devices. Repeatability means the absence of incompatibility between consecutive measurements by the same electrode, whilst reproducibility is the vicinity of the results acquired by means of a number of the same modified electrodes with the same measurement procedure. The CV responses of three different electrodes with successive addition of 5 mM glucose were recorded at the optimal condition. The relative standard deviation (RSD) of the response currents for different CuO/ZnO-DSDSHNM electrodes was only 1.3%, suggesting good reproducibility and precision (Fig. 9a). The repeatability was examined by three times of measurements by a single modified GCE in 1 day. The RSD of the repeatability was calculated as 2.23% as shown in Fig. 9b, which shows the excellent repeatability of the proposed sensor. The selectivity of CuO/ZnO-DSDSHNM for glucose sensing was evaluated by the addition of some potential interferences when conducting a typical amperometric detection. As shown in Fig. 9c, an obvious steady-state current appeared upon addition of 5 mM glucose at 0.6 V. In addition, no current change was observed with the additions of 0.1 mM ascorbic acid (AA), 0.1 mM uric acid (UA), 0.1 mM dopamine (DA), 0.1 mM Cl^−^, 0.1 mM Na^+^, 0.1 mM lactose and 0.1 mM sucrose. This insensible behavior followed by a dramatic current increase in response to 5 mM glucose suggests the high selectivity of CuO/ZnO-DSDSHNM for glucose sensing. Long-term stability and lifetime of the electrode were investigated by measuring the electrode response in the presence of 5 mM glucose in 0.50 M NaOH solution at a scan rate of 50 mVs^−1^. The stability of the proposed electrochemical sensor was evaluated by storing the Nafion/CuO/ZnO-DSDSHNM modified GCE electrodes in air and recording the current response of 5 mM glucose. Figure 9d showed that the response current of 5 mM glucose could retain about 92.88% of its initial value after storing Nafion/CuO/ZnO-DSDSHNM modified GCEs in the air for 15 days, indicating excellent storage stability.

### Glucose detection in real human blood

Analysis of non-diabetic human blood serum samples was performed to evaluate the commercial viability of the developed non-enzymatic Nafion/CuO/ZnO-DSDSHNM/GCE glucose sensor. Additionally, these real blood samples were analyzed using the DIRUI CS-800 Auto Chemistry Analyzer device for validating the results. The amperometric analysis was performed by injection of 30 µl of the sample into the 30 ml of 0.50 M NaOH at the potential of + 0.60 V (versus Ag/AgCl). The analysis was performed via the standard addition method. The same aliquots of serum samples were spiked by the known concentrations of glucose and 30 µl of each sample was injected into the 30 ml of NaOH solution. The electrochemical analysis of each sample was done by using Nafion/CuO/ZnO-DSDSHNM/GCE three times. Anywhere which was necessary, the volume correction was performed. The Nafion/CuO/ZnO-DSDSHNM/GCE glucose sensor electrode provided an acceptable recovery ranging from 95.84 to 104.83% for the human blood serum samples as shown in Table 2.

**Table 2:** Recovery and relative standard deviation (RSD) of Nafion/CuO/ZnO-DSDSHNM/GCE for detecting glucose in a real serum sample.

Sample (mM)	Founded (mM)	RSD (%)	Recovery (%)
2.57	2.63	0.12	102.33
4.09	3.92	0.97	95.84
5.80	6.08	0.1	104.83
8.47	8.63	0.4	101.89

## Conclusion

A new double-shelled hollow nanoporous CuO/ZnO microstructure was successfully fabricated by a hydrothermal method, designed for non-enzymatic electrochemical detection of glucose. As a result, an efficient and rapid electrochemical glucose biosensor was fabricated and optimized. The results revealed that the sensor optimizations performed have been successful and the proposed sensor showed a wide dynamic detection range of 500 nM–100 mM, remarkable sensitivity of 1536.8 ± 79.6 µA mM^−1^ cm^−2^, and very low LOD of 357.5 nM. The Nafion/CuO/ZnO-DSDSHNM modified GCE exhibited excellent reproducibility and repeatability, favorable stability, and satisfactory selectivity toward glucose sensing in a 0.5 M NaOH solution. The cost-effective synthesis procedure and the outstanding catalytic performance suggest that the CuO/ZnO-DSDSHNM material may be promising for non-enzymatic glucose sensing in practical analysis applications.

## Methods

### Materials

Zinc Nitrate (Zn(NO_3_)_2_), Copper Nitrate (Cu(NO_3_)_2_), Sodium Hydroxide (NaOH), Aluminum Oxide (Al_2_O_3_), AA, DA, lactose, sucrose, NaCl, Nafion, glucose, and pluronic F-127 were purchased from Sigma-Aldrich, Merck and DuPont and human blood serum was taken from the hospital. In this study, all the materials were of analytical grade and used as received without any further purification. All solutions were prepared by DI water (3 μS/cm) generated from the Deltino water purifying system.

### Apparatus

XRD was taken by BRUKER D8 Advanced. SEM and elemental mapping data were obtained by using TESCAN Mira3. The TEM images were taken with digital charge-coupled device JEOL-JEM-2010 equipped with a CCD camera. XPS experiments were measured using an ESCAlab 250 Analytical XPL Spectrometer with a monochromatic Al Ka source. ICP-AES test was performed by ICP-MS ELANDRC-E Perkin-Elmer.The Electrochemical measurements were performed by using IVIUM Vertex potentiostat/galvanostat electrochemical analyzer. All experiments were conducted using a three-electrode system with a glassy carbon-based working electrode (2 mm diameter), an Ag/AgCl reference electrode (3 M KCl), and a platinum wire for the counter electrode (1 cm length and 1 mm diameter). The CVs and choronoamperometric measurements were performed with an electrochemical analyzer in a 0.50 M NaOH solution.

### Synthesis of materials

Three types of hollow structured materials have been synthesized. As the main material, a unique dumbbell-shaped double-shelled hollow nanoporous CuO/ZnO microstructures was synthesized. For comparison of electrocatalytic activity, the CuO-MS and ZnO-MS also were synthesized via a similar method. The procedure for synthesizing the above-mentioned materials are as follows:

First, 3 g of glucose, 1 g of F-127 as a surfactant were dissolved in 150 ml deionized water and the solution was sonicated for 10 min. Then, 2 ml of 1 M of Zn(NO_3_)_2_ solution and 2 ml of 1 M of Cu(NO_3_)_2_ solution were added to the mixture. The mixture of glucose, surfactant and both metal salts was placed into the autoclave and the hydrothermal process was directed for 24 h at 180 ֯C. The resulted product was washed three times with distilled water and dried in an oven at 80 ֯C overnight. The final structure was achieved via the calcination of the product from the hydrothermal step at 550 ֯C for 270 min.

The synthesis of CuO-MS and ZnO-MS was achieved via the same procedure with the same amount of reagents but by using the single salt of each metal at processes.

### Electrode preparation (Nafion/CuO/ZnO-DSDSHNM/GCE)

At first step, the GCE was polished by means of alumina slurry over a Pad, and washed three times with DI water and ethanol, respectively. To fabricate the Nafion/CuO/ZnO-DSDSHNM/GCE, the solution of 5 mg/ml of synthesized material in ethanol was prepared and ultrasonically homogenized for 20 min. After that, 2 μl of CuO/ZnO-DSDSHNM solution was casted on the surface of GCE until it dried at room temperature. Next, 2 μl of 0.5 wt% Nafion solution was casted on the catalyst layer at GCE and then dried at ambient conditions. Henceforth, the Nafion/CuO/ZnO-DSDSHNM/GCE electrode was prepared for performing electrochemical measurements.

### Statement

All experiments and methods were performed in accordance with relevant guidelines and regulations. All experimental protocols were approved by the ethics committee of the laboratory of Shahid Beheshti Hospital. Informed consent was obtained from all subjects.

## Supplementary Information

Supplementary Information 1.
