# Supplementary material for: Highly sensitive non-enzymatic electrochemical glucose sensor based on dumbbell-shaped double-shelled hollow nanoporous CuO/ZnO microstructures
Source: Sci Rep. 2021 Jan 11;11:344. doi: 10.1038/s41598-020-79460-2 (PMC7801383; doi:10.1038/s41598-020-79460-2)
Supplement: Supplementary file 1 — Supplementary Information 1. [file 41598_2020_79460_MOESM1_ESM.docx]

**Supplementary Information**

**Highly Sensitive Non-Enzymatic Electrochemical Glucose Sensor Based on Dumbbell-Shaped Double-Shelled Hollow Nanoporous CuO/ZnO Microstructures**

Zahra Haghparas,^a^ Zoheir Kordrostami,^a,*^ Mohsen Sorouri,^b,*^ Maryam Rajabzadeh,^b^ Reza Khalifeh,^b^

^a^Department of Electrical and Electronic Engineering, Shiraz University of Technology, Shiraz, Iran

^b^Department of chemistry, Shiraz University of Technology, Shiraz, Iran

***Corresponding authors.** E-mail: [kordrostami@sutech.ac.ir](mailto:kordrostami@sutech.ac.ir) , sorouri@sutech.ac.ir

The GCE was modified with different concentrations of CuO/ZnO-DSDSHNM. Similar to the electrochemical procedure in the article, CV tests were performed using 0.5 M of NaOH solution and 5 mM glucose at a scan rate of 50 mVs^-1^. The effect of the CuO/ZnO-DSDSHNM concentration on the current is shown in figure S1. Accordingly, the highest amount of current was achieved using 5 mg/ml of electrocatalyst for electrode modification.


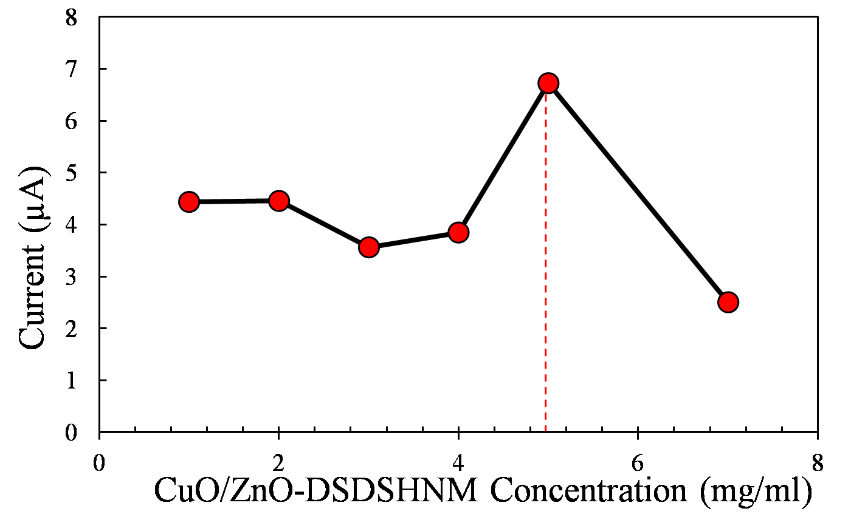


Figure S1. The current at 0.6 V versus different concentrations of casted CuO/ZnO-DSDSHNM (1, 2, 3, 4, 5, and 7 mg/ml) in 0.50 M NaOH solution with 5 mM glucose at a scan rate of 50 mVs^-1^.

Afterward, the Nafion concentration was optimized. To this purpose, after casting 5 mg/ml of electrocatalyst, the modified GCE was coated with different concentrations of Nafion. The currents versus the Nafion concentration have been shown in figure S2. As it is observable, the optimum amount of Nafion concentration was 0.50 wt%.


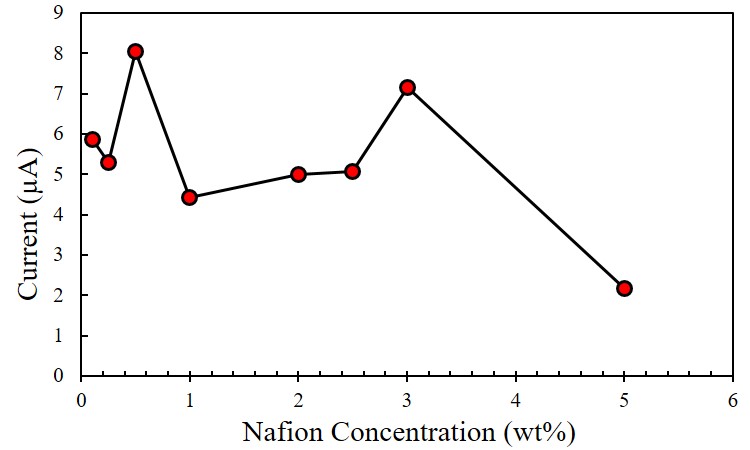


Figure S2. The current at 0.6 V versus different concentration of Nafion (0.10, 0.25, 0.50, 1, 2, 2.5, 3 and 5 wt%) in 0.50 M NaOH solution with 5 mM glucose at scan rate of 50 mVs^-1^.

The electrochemical test conditions should be also optimized. At first, several voltage ranges were investigated. As can be seen in figure S3, the best response to was obtained when setting the CV range between 0 and 0.80 V.


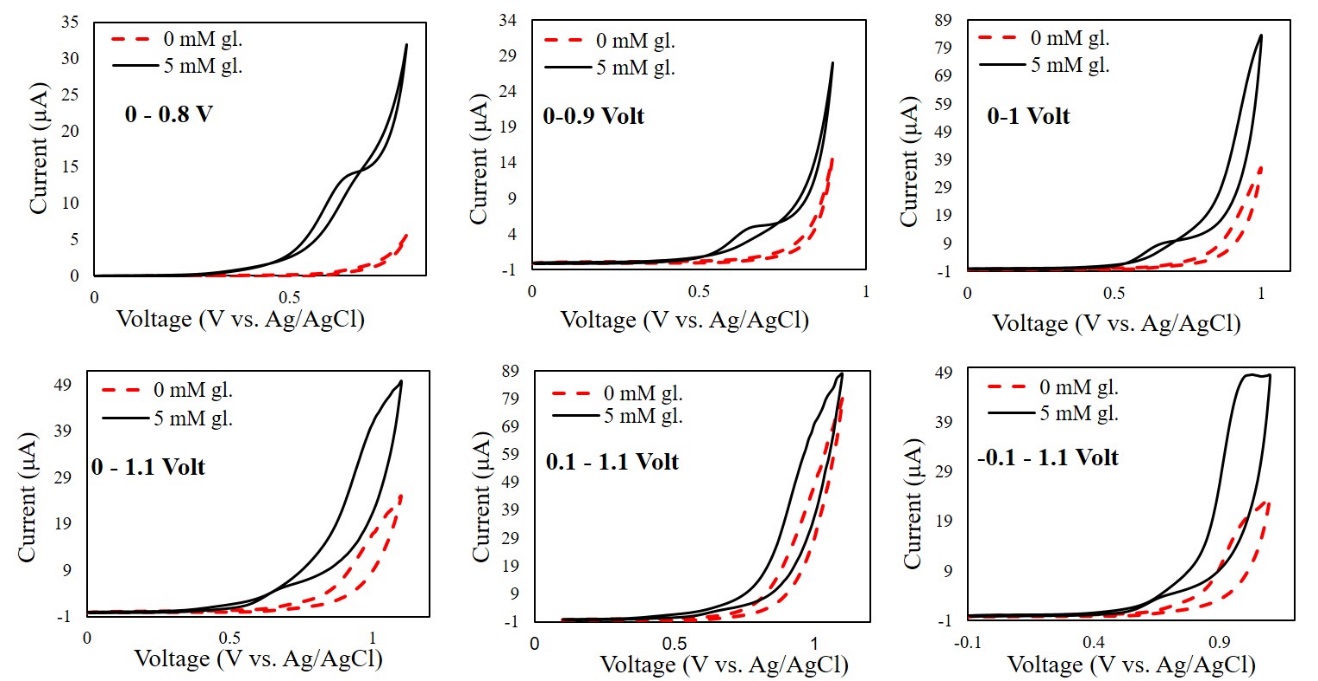


Figure S3. The CVs responses of Nafion/CuO/ZnO-DSDSHNM/GCE at different voltage ranges ((0-0.80 V), (0-0.90 V), (0-1 V), (0-1.10V), (0.10-1.10 V) and (-0.10-1.10 V)) in 0.50 M NaOH solution with 0 and 5 mM glucose concentration.

Next, the behavior of the proposed sensor was investigated in several NaOH concentrations containing 0.05 M, 0.10 M, 0.50 M, and 1 M. The CVs for different concentrations of NaOH as supporting electrolyte are shown in figure S4. Among them, the best performance was obtained for 0.50 M NaOH solution.


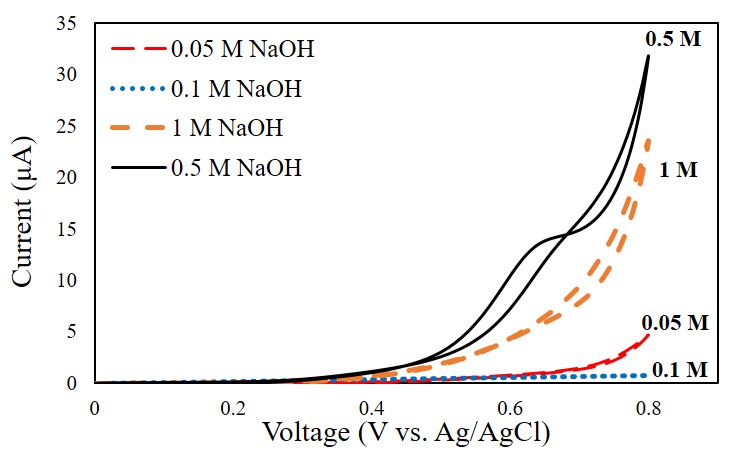


Figure S4. The CV responses of Nafion/CuO/ZnO-DSDSHNM/GCE in different concentrations of NaOH (0.05, 0.10, 0.50, and 1 M) at the presence of 5 mM glucose.

The optimum potential of the amperometry test was investigated by recording the corresponding amperogram at different voltages (0.45, 0.50, 0.55, 0.60, 0.65, and 0.70 V). Figure S5 indicates i-t curves in the mentioned voltages. Accordingly, the maximum current for glucose oxidation has occurred at the oxidation potential of 0.60 V.


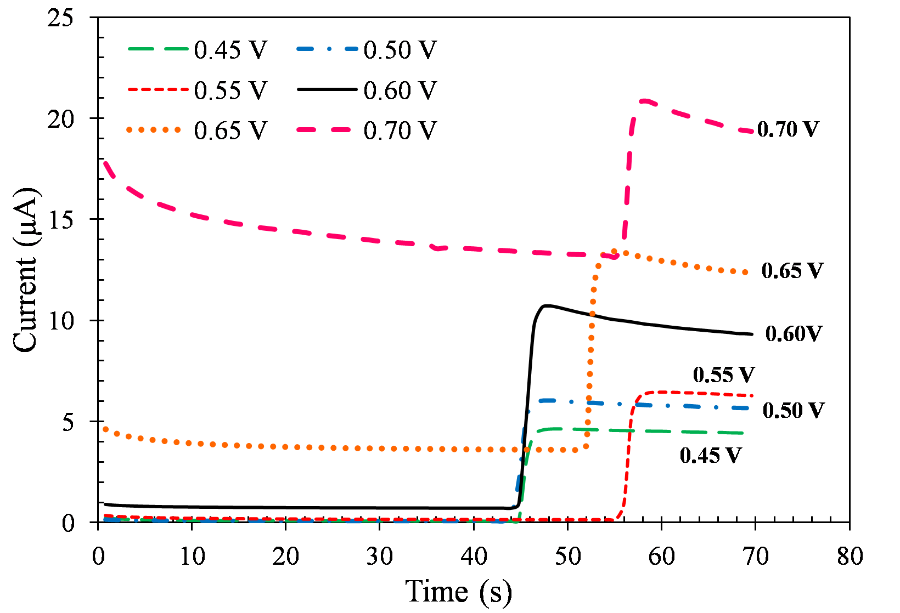


Figure S5. The amperograms of modified GCE at different voltages (0.45, 0.50, 0.55, 0.60, 0.65 and 0.70 V) in 0.50 M NaOH solution at the presence of 5 mM glucose.

Figure S6 represents the calibration plot of the i-t curve for Nafion/CuO/ZnO-DSDSHNM/GCE which can be described by a power plot with the following regression equation: I(µA) = 0.966C^0.264^(µM) (R² = 0.9656). The corresponding calibration curve could be broken into three linear ranges which are discussed in the manuscript in detail.


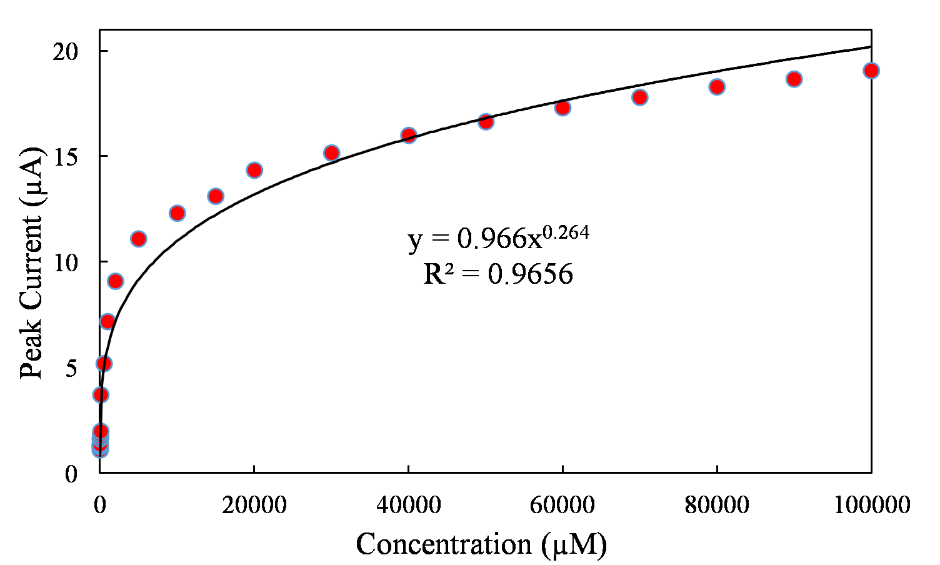


Figure S6. Calibration plot of the current response as a function of the glucose concentration over the range from 500 nM to 100 mM fitting by power plot.
